# Pregnancy outcomes in women at high risk of preterm birth receiving a vaginal cervical cerclage with, or without, progesterone: A retrospective, secondary analysis of the C-STICH randomised controlled trial data

**DOI:** 10.1371/journal.pmed.1004513

**Published:** 2026-01-23

**Authors:** Victoria Hodgetts Morton, Katie Morris, Philip Toozs-Hobson, Lee Middleton, Nicole Pilarski, Lilah Bell, Martha Hogg, Rebecca Man, Fidan Israfil-Bayli, Andrew Shennan, Nigel Simpson, Christoph Lees, Catherine Moakes

**Affiliations:** 1 Department of Applied Health Sciences, University of Birmingham, Birmingham, United Kingdom; 2 Department of Obstetrics and Gynaecology, Birmingham Women’s Hospital, Birmingham, United Kingdom; 3 College of Medical and Dental Sciences, University of Birmingham, Birmingham, United Kingdom; 4 Department of Obstetrics and Gynaecology, King’s College London, London, United Kingdom; 5 Leeds Institute for Medical Research, University of Leeds, Leeds, United Kingdom; 6 Department of Metabolism, Digestion and Reproduction, Imperial College London, London, United Kingdom; Makerere University College of Health Sciences, UGANDA

## Abstract

**Background:**

Vaginal cervical cerclage and progesterone are established treatments for prevention of pregnancy loss and prematurity. There is limited data to assess the effect of these treatments in combination. The objective of this study was to investigate the association between progesterone and no progesterone treatment on pregnancy outcomes in women at high risk of preterm birth who had received a vaginal cervical cerclage.

**Methods and findings:**

This is a secondary post-hoc analysis of women recruited to the C-STICH randomised controlled trial, which recruited in 75 obstetric units in the UK between 2015 and 2021. In the C-STICH trial, women with a singleton pregnancy, receiving a vaginal cervical cerclage due to a history of pregnancy loss or premature birth, or if indicated by ultrasound, were randomised to cerclage with braided or monofilament suture, with a primary outcome of pregnancy loss, defined as miscarriage, stillbirth, or neonatal death in the first week of life. In this secondary analysis, the primary outcome was pregnancy loss, defined as miscarriage and perinatal mortality, including any stillbirth or neonatal death in the first week of life. Secondary maternal outcomes included miscarriage and previable neonatal death; stillbirth; gestational age at delivery; preterm pre labour rupture of membranes, and sepsis. Secondary neonatal outcomes included early/late neonatal death and sepsis. For each outcome, regression models were fitted adjusting for prespecified prognostic variables.

From the 2,048 women recruited to C-STICH, 1943 (95%) women had a vaginal cerclage placed and available progesterone data. Of these, 834 (43%) women received progesterone and 1,109 (57%) did not receive progesterone. In women with primary outcome data available, in our predefined analysis pregnancy loss occurred in 49 (5.9%) of 832 women who received progesterone and 91 (8.3%) of 1,103 women who did not receive progesterone (adjusted* risk ratio 0.70 (95% confidence interval (CI) [0.50, 0.99]); adjusted risk difference −0.02 (95% CI [−0.04, −0.001], *adjusted for indication, obstetric history, surgical technique, and maternal age). Further exploratory analysis excluding women who had termination of pregnancy for foetal anomaly demonstrated a nonsignificant reduction in the risk of pregnancy loss. Key limitations of this study include a nonrandomised trial design and unknown confounding relating to variation in progesterone use.

**Conclusion:**

In women with a vaginal cervical cerclage and concomitant progesterone there appears to be an association with a reduced risk of pregnancy loss. This combination therapy may be an important opportunity to further reduce the risk of pregnancy loss in this high-risk cohort.

## Author summary

**Why was this study done?:**

- We aimed to evaluate whether the risk of pregnancy loss in women at high risk of premature birth with a vaginal cervical suture in place is different with and without progesterone treatment.

**What did the researchers do and find?:**

- The researchers completed a secondary analysis of the data from C-STICH, a randomised controlled trial of different suture types for vaginal cervical cerclage, to compare outcomes pregnancy outcomes with and without additional progesterone therapy.
- Use of progesterone and vaginal cerclage was associated with a statistically significant reduction in the risk of pregnancy loss in an adjusted analysis (5.9% versus 8.3%).

**What do these findings mean?:**

- In women at high risk of preterm birth, combination therapy with vaginal cervical cerclage, and progesterone is associated with a statistically significant reduction in the risk of pregnancy loss.
- This analysis benefits from the large population recruited to the C-STICH trial but interpretation is limited by the available details regarding timing and indications for progesterone use.
- A future randomised trial is not realistic, however, progesterone is a widely accepted therapy for prevention of preterm birth, and this research provides new evidence to guide the clinical management of women at high risk of preterm birth.

## Abstract 3

In a secondary analysis of the CSTICH trial, Victoria Hodgetts Morton and colleagues investigate the association between progesterone treatment versus no treatment on pregnancy outcomes in women at high risk of preterm birth who had received a vaginal cervical cerclage.

## Introduction

Second trimester miscarriage and preterm birth is a multifactorial condition affecting ~10% of pregnancies. Second trimester miscarriage and preterm birth are a continuum of conditions with overlapping aetiology [1]. Miscarriage is defined as pregnancy loss prior to 24 weeks gestation and in the C-STICH study this included neonatal deaths of babies born prior to 24 weeks gestation. Preterm birth can be defined as extreme (<28 weeks), very (28–32 weeks gestation), or late prematurity (32–37 weeks gestation). The prematurity syndrome does not have a singular cause with cervical weakness, inflammation and infection, stress, uterine overdistension, and placental/vascular disorders all contributing to a poorly understood process that potentially prematurely initiates the labour process [1].

Vaginal cervical cerclage and vaginal progesterone are both established treatments to reduce the risk of second trimester miscarriage and preterm birth [2,3]. The evidence for both progesterone and vaginal cervical cerclage has been ascertained from independent interventions in the same population of women at high risk of pregnancy loss [4,5]. It is therefore reasonable to consider whether these treatments have benefit in combination. The evidence for the use of vaginal progesterone and cervical cerclage in combination is limited and there are no randomised controlled trials designed specifically to answer this question. A retrospective cohort of 699 women with a cerclage investigated concurrent treatment and identified a reduction in prematurity in those women with concurrent progesterone [6]. Additionally, Roman and colleagues described the potential benefit of rescue vaginal progesterone in women who had continued cervical shortening despite the placement of a vaginal cervical cerclage, demonstrating a potential prolongation of pregnancy (36.36 weeks versus 32.63 weeks; *p* = 0.0036) [7]. This was a small study consisting of 66 women but highlights the need to consider whether these treatments provided additional benefit when used in combination.

C-STICH was a randomised controlled trial of type of suture thread (monofilament versus braided) in women undergoing a vaginal cervical cerclage [8]. C-STICH recruited 2,048 eligible women where the clinical decision for a vaginal cervical cerclage had already been made. Women were then randomised prior to the cervical cerclage surgery to either a monofilament or braided suture thread. The trial was pragmatic in that the operative technique/adjuncts, decision to use progesterone and clinical management were all at the discretion of the local recruiting clinical team. Within C-STICH there was no difference in the primary outcome of pregnancy loss (defined as miscarriage and perinatal mortality, including any stillbirth or neonatal death in the first week of life) [9]. The data collected within C-STICH has provided the opportunity to examine the effect of receiving progesterone on pregnancy loss, prematurity and maternal and neonatal outcomes, in women who undergo a vaginal cervical cerclage. The pragmatic nature of the trial means that we have variation in practice regarding progesterone use in women undergoing cervical cerclage. National UK guidelines recommend the use of either progesterone or cervical cerclage in women with a risk factor for preterm birth and a short cervix and there is no clear recommendation regarding combination treatment [9]. The trial was conducted between 2015 and 2021, and during this time there was additional data published regarding the effectiveness of progesterone and a shift towards increasing progesterone use [10].

The aim of this analysis was to examine the effect of combination treatment of progesterone and vaginal cervical cerclage, compared with vaginal cervical cerclage alone on pregnancy outcomes in women at high risk of preterm birth.

The primary objective was to investigate whether vaginal progesterone reduces the risk of pregnancy loss, in women with a vaginal cervical cerclage due to an increased risk of preterm birth. Secondary objectives were to assess the characteristics of women (including indication for cerclage) who did or did not receive progesterone and to assess whether in women with a vaginal cervical cerclage, vaginal progesterone improves other pregnancy and neonatal outcomes [8].

## Methods

This was a secondary analysis of participants in the C-STICH trial (ISRCTN15373349) which recruited in 75 obstetric units in the UK and was published previously [9]. The protocol for the primary randomised controlled trial was published prior to the trial [8]. The C-STICH trial was a 1:1 randomised, pragmatic, superiority trial of monofilament compared to braided cervical cerclage. Women in the original randomised controlled trial were centrally randomised by Birmingham Clinical Trials Unit, using minimisation, with women and assessors masked to allocation. No ethical approval was required for the secondary analysis of this data, ethical approval for the original trial was obtained [8].

### Patients

Women were considered eligible for the C-STICH trial if they had an indication for a vaginal cervical cerclage, were aged 18 years or older and had a singleton pregnancy. Indication for cervical cerclage was defined as either a history of three or more previous midterm losses or premature births (≤28 weeks), insertion of cervical sutures in previous pregnancies, a history of midtrimester loss or premature birth, with a shortened cervix (≤25 mm) in the current pregnancy, or clinician concern for risk of preterm birth either due to history or the results of an ultrasound scan. Women were ineligible if they required an emergency or rescue cerclage; needed immediate insertion of a suture; had membranes that had ruptured or were visible or required a cerclage which was to be placed by any route other than vaginally (e.g., via an abdominal route). All participants provided written informed consent. The trial was pragmatic and aimed for recruitment of the majority of women undergoing a vaginal cervical cerclage within the UK at the time. There was no exclusion based on concomitant treatments such as antibiotics, progesterone, and surgical technique. For this secondary analysis women had to have available progesterone data and had a vaginal cervical cerclage successfully placed. A statistical analysis plan was developed for this secondary analysis following the principles of the randomised controlled trial with outcomes derived as per the original analysis, this was agreed within the C-STICH team before this analysis was performed.

The intervention group received combination treatment of progesterone and vaginal cervical cerclage. Treatment of progesterone was defined as any progesterone use in pregnancy. The comparator group received vaginal cervical cerclage alone.

### Outcomes

The primary outcome is pregnancy loss rate defined as miscarriage and perinatal mortality, including any stillbirth or neonatal death in the first week of life. A key secondary outcome was time from conception to pregnancy end. Secondary outcomes included the following maternal and neonatal outcomes: miscarriage and previable neonatal death (defined as delivery <24 weeks); stillbirth (defined as intrauterine death ≥24 weeks); gestational age at delivery (in live births ≥24 weeks); gestational age <28/<32/<37 weeks at delivery (in live births ≥24 weeks); preterm prelabour rupture of membranes (PPROM); maternal sepsis (at any time in pregnancy and until 7 days postnatal); early neonatal death (defined as a death within 7 days after delivery, in live births ≥24 weeks); late neonatal death (defined as a death beyond 7 days and before 28 days after delivery, in live births ≥24 weeks), and neonatal sepsis (clinically diagnosed/proven) (in live births ≥24 weeks).

### Sample size

There were 1,943 participants within C-STICH who had a vaginal cerclage successfully placed and progesterone data were available. Whilst this sample size was not specifically tailored to this secondary analysis, this size of sample would be sufficient to detect a reduction in pregnancy loss from 9.3% in the combination treatment of progesterone and vaginal cervical cerclage group, to 5.7% in the vaginal cervical cerclage only group, with 85% power (two-sided, α = 0.05).

### Statistical analysis

A statistical analysis plan was prespecified before analysis attached as supplementary file (S1 Text). All estimates of differences between groups were analysed using regression models presented with two-sided 95% confidence intervals (CIs) adjusted for primary indication for cerclage (a history of three or more previous midterm losses or premature births (≤28 weeks)/insertion of cervical sutures in previous pregnancies/a history of midtrimester loss or premature birth with a (current) shortened (≤25 mm) cervix/women whom clinicians deem to be at risk of preterm birth either by history or the results of an ultrasound scan); number of previous midtrimester losses; number of previous preterm births (<34 weeks); any previous cervical surgery (yes/no/unknown); cerclage technique involved bladder dissection (yes/no); suture type received (monofilament/braided); ethnicity (White/Black/Asian/mixed/other/declined to provided information), and maternal age at cerclage placement (where possible). Time from conception to live birth and gestational age at delivery were further adjusted for gestational age at cerclage placement. If full covariate adjustment was not possible (e.g., the model did not converge), covariates were removed sequentially in the reverse order to those listed above, with the exception of issues related to collinearity, whereby the affected covariate was removed first. The variables for adjustment within this analysis are consistent with the randomisation minimisation within the primary randomised controlled trial (primary indication for cerclage and cerclage technique involved bladder dissection) which was determined by an expert group to have a significant effect on the overall risk of pregnancy loss. In addition to the expert opinion from the C-STICH collaborators, a literature review of prognostic factors for success of cerclage identified the additional variables included above the minimisation variables [10,11].

Baseline data were summarised descriptively and included those listed above as adjustment covariates. Tests of statistical significance were undertaken using *t*-tests or Wilcoxon rank sum tests for continuous variables (depending on the distribution of the data) and chi-squared or Fisher’s exact tests for categorical variables.

The primary outcome was summarised using frequencies and percentages. A log-binomial model was used to generate an adjusted risk ratio (RR) (and 95% CI). An adjusted risk difference (RD) (and 95% CI) was also presented (using an identity link function). Binary secondary outcomes (miscarriage and previable neonatal death, stillbirth, gestational age<28/<32/<37 weeks, PPROM, and early neonatal death and late neonatal death) were analysed as per the primary outcome. Continuous secondary outcome measures (gestational age at delivery) were summarised using means and standard deviations alongside an adjusted mean difference (with 95% CI) estimated using a linear regression model.

Time to event data (time from conception to pregnancy end) were considered using traditional time-to-event analysis methods (Cox regression) and in a competing risk framework (time from conception to live birth). A competing risk framework was considered to account for the different outcomes of pregnancy end. A cumulative incidence function was used to estimate the probability of a live birth (surviving at least 7 days) over time, accounting for the competing event of pregnancy loss. This method is favoured over Kaplan–Meier methods which may overestimate this probability. A Fine-Grey model was used to estimate a sub distribution adjusted hazard ratio (HR) (and 95% CI) directly from the cumulative incidence function. The sub distribution HR estimates the overall incidence of live birth (i.e., presence of live birth over time). In addition, a Cox Proportional Hazard model was fitted and applied to the cause-specific hazard function and used to generate a cause-specific adjusted HR (and 95% CI) which estimates the rate in which live births occur [12,13].

Since the proportion of participants with missing data for the primary outcome was <1% (*N* = 8), no supporting analyses to assess the impact of missing data were conducted (i.e., complete case analyses only).

We conducted prespecified subgroup analyses (limited to the primary outcome measure only) for number of previous midtrimester losses (<3/≥3) and number of previous preterm births (<34 weeks) (0/≥1). The effects of these subgroups were examined by adding the subgroup by treatment group interaction parameters to the regression model. *P*-values from the tests for statistical heterogeneity were presented with the effect estimate and estimates of uncertainty within each subgroup. Additionally, ratios were provided to quantify the difference between the treatment effects estimated within each subgroup.

All analyses were performed in SAS (version 9.4) or Stata (version 18.0).

## Results

Of the 2,048 women randomised to C-STICH (screened for eligibility between 2015 and 2021), 1,998 women had a cerclage placed, with progesterone data available in 1,943 women. Of these, 834 (43%) received progesterone treatment in combination with their vaginal cervical cerclage. Progesterone was commenced greater than 7 days prior to cerclage placement in 191 (25%) of cases, commenced within a week of cerclage placement in 298 (39%) of cases and greater than 7 days after cerclage placement in 277 (36%) of cases (detailed in Table 1).

**Table 1:** Summary of progesterone use in the C-STICH population.

	Number randomised to the C-STICH trial (*N* = 2,048^*^)
Cerclage placed-*N* (%)	
Yes	1998 (98)
No	45 (2)
Missing	5
Progesterone use^1^-*N* (%)	
Yes	834 (43)
No	1,109 (57)
Missing	55
Gestational age progesterone received (weeks)^2^	
Median [IQR, N]	16.0 [13.6–19.1, 766]
Minimum–Maximum	0–34.6
Missing	68
Time from cerclage placement to progesterone start (days)^2^-*N* (%)	
≥7 days prior to cerclage placement	191 (25)
<7 days prior to cerclage placement or <7 days after cerclage placement	298 (39)
≥7 days after cerclage placement	277 (36)
Median [IQR, N]	1.0 [−6 to 13, 766]
Minimum–Maximum	−133–151
Missing	68
Progesterone type^2^^,^^3^-*N* (%)	
Cyclogest 200 mg PV daily	239 (29)
Cyclogest 400 mg PV daily	429 (53)
17 alpha-hydroxyprogesterone 250 mg IM weekly	117 (14)
Other	71 (9)
Missing	22

A summary of the data available for progesterone usage among women participating in the C-STICH trial. Abbreviations: IQR: interquartile range; PV: per vagina; IM: intramuscular.

*Excluding the woman who was randomised in error as no data was collected.

^1^In women who had a cerclage placed.

^2^In women who had a cerclage place and received progesterone.

^3^Progesterone types are not mutually exclusive.

The demographics of the included participants are detailed in Table 2. There were notable differences between those that did not receive additional progesterone treatment and those who did. Women without any previous premature live births accounted for the majority of the study population in both groups however they were more likely to receive combined treatment than women who had previously had liveborn preterm babies. Women who underwent a cervical cerclage with bladder dissection were also more likely to receive combined treatment—in women with a cerclage with bladder dissection 17% did not receive progesterone and 34% received progesterone (*p* < 0.01). In addition, those women who were non-black ethnicity (78% in those who did not receive progesterone versus 85% in those who received progesterone, *p* = 0.01) and women whose indication for cerclage was due to risk of preterm birth through history or ultrasound (58% in those who did not receive progesterone versus 63% in those who received progesterone, *p* = 0.03). The median gestational age at cerclage placement was 16.3 weeks in those who did not receive progesterone and 15.9 weeks in those who received progesterone (*p* = 0.02).

**Table 2:** Summary of characteristics in the C-STICH population by progesterone use.

	Received progesterone		Estimate (95% CI) *p*-value^*^
	No (*N* = 1,109)	Yes (*N* = 834)	
Suture type received-*N* (%)			
Monofilament suture	553 (50)	408 (49)	1.02 (0.93, 1.11)^1^
Braided suture	556 (50)	426 (51)	0.68
**Participant characteristics**			
Gestational age at cerclage placement (weeks)			
Median [IQR, N]	16.3 [14.1–19.6, 1,109]	15.9 [13.7–19.4, 834]	−0.43 (−1.00, 0.14)²
Minimum–Maximum	8.3–25.7	5.1–28.6	0.02
Maternal age at cerclage placement (years)			
Mean (SD, N)	32.7 (4.9, 1,109)	33.2 (5.1, 834)	0.43 (−0.02, 0.88)³
Minimum–Maximum	18.2–52.3	18.6–48.3	0.06
Ethnicity-*N* (%)			
White	600 (55)	486 (59)	1.05 (0.83,1.33)^4^
Asian	207 (19)	176 (21)	0.66 (0.51, 0.84)^4^
Black	239 (22)	127 (15)	1.01 (0.63,1.64)^4^
Mixed	39 (3)	32 (4)	0.71 (0.29, 1.70)^4^
Other	14 (1)	8 (1)	0.01
Missing	10	5	–
**Pregnancy history**			
Number of previous live preterm births-*N* (%)			
0	726 (65)	582 (70)	–
1	310 (28)	206 (25)	
2	61 (6)	39 (5)	
≥3	12 (1)	7 (<1)	
Median [IQR, N]	0 [0–1, 1,109]	0 [0–1, 834]	0.00 (-)^5^ 0.04
Number of previous midtrimester losses-*N* (%)			
0	528 (48)	360 (43)	–
1	391 (35)	344 (41)	
2	153 (14)	104 (13)	
≥3	37 (3)	26 (3)	
Median [IQR, N]	1 [0–1, 1,109]	1 [0–1, 834]	0.00 (−0.42, 0.42)^2^ 0.24
**Clinical characteristics**			
Primary indication for cerclage**-***N* (%)			
Deemed risk of preterm birth through history or ultrasound	642 (58)	522 (63)	0.93 (0.74, 1.16)^6^
Insertion of cervical sutures in previous pregnancies	237 (21)	179 (21)	0.75 (0.58, 0.96)^6^
History of midtrimester loss/premature birth + shortened cervix	198 (18)	120 (14)	0.50 (0.26, 0.96)^6^
History of ≥3 previous midterm losses/premature births	32 (3)	13 (2)	0.03
Cerclage technique include bladder dissection**-***N* (%)	188 (17)	285 (34)	2.02 (1.72, 2.38)^7^ <0.01
Missing	0	3	–
Previous cervical surgery**-***N* (%)	285 (26)	227 (27)	1.06 (0.91, 1.23)^7^ 0.44
Missing	2	2	–

This table presents the population characteristics of the C-STICH study by progesterone use, including estimate of the relative difference, 95% confidence interval and *p*-value between progesterone groups for each characteristic. Abbreviations: IQR: interquartile range; SD: standard deviation.

* Estimate (received progesterone versus no progesterone) and *p*-value derived from univariate models/tests.

^1^Risk ratio (braided suture).

^2^Difference in medians.

^3^Mean difference.

^4^Risk ratio (Asian, Black, Mixed, and Other).

^5^Difference in medians, 95% CI not computed as estimated bootstrap variance was zero.

^6^Risk ratio (insertion of cervical sutures in previous pregnancies, History of midtrimester loss/premature birth + shortened cervix, and history of ≥3 previous midterm losses/premature births).

^7^Risk ratio (Yes).

Pregnancy loss occurred in 49 (5.9%) of 832 women who received progesterone and 91 (8.3%) of 1,103 women who did not receive progesterone (adjusted risk ratio 0.70 (95% CI [0.50, 0.99]); adjusted risk difference −0.02 (95% CI [−0.04, −0.001]) as per Table 3. On reviewing this analysis, there were 6 terminations of pregnancy in the no progesterone group for foetal anomaly compared to 1 in the progesterone group and therefore an unplanned sensitivity analysis was performed removing terminations for foetal anomalies from the definition of pregnancy loss (the primary outcome). Within the sensitivity analysis, pregnancy loss occurred in 48 (5.8%) of 832 women who received progesterone compared to 85 (7.7%) of 1,103 women who did not receive progesterone (adjusted risk ratio 0.74 (95% CI [0.53, 1.05]); adjusted risk difference −0.02 (95% CI [−0.04, 0.004]) as per Table 4.

**Table 3:** Primary analysis of pregnancy loss in the C-STICH population with and without progesterone.

	Received progesterone		Risk ratio^1^ (95% CI)	Risk difference^2^ (95% CI)
	No (*N* = 1,109)	Yes (*N* = 834)		
Pregnancy loss-*N* (%)				
Yes	91 (8.3)	49 (5.9)	0.70 (0.50, 0.99)	−0.02 (−0.04, −0.001)
No	1,012 (91.8)	783 (94.1)		
Missing	6	2		
Type of pregnancy loss-*N* (%)				
Miscarriage	48 (4)	22 (3)	–	–
Spontaneous	45	20		
Missed	1	1		
Septic	2	1		
Termination	16 (1)	4 (<1)		
Foetal anomaly	6	1		
Maternal medical condition	1	0		
Maternal sepsis	7	1		
Other^3^	2	2		
Stillbirth	8 (1)	10 (1)		
Other^4^	1 (<1)	0 (-)		
Neonatal death <7 days	18 (2)	13 (2)		

Pregnancy loss (primary outcome) presented by progesterone group including adjusted risk ratio with 95% confidence intervals and adjusted risk difference with 95% confidence intervals. Progesterone use by pregnancy loss type is also presented without further analysis. Abbreviation: CI: confidence interval.

^1^Adjusted for primary indication for cerclage, number of previous midtrimester losses, number of previous preterm births (<34 weeks), previous cervical surgery, cerclage technique involved bladder dissection, and suture type received and maternal age at cerclage placement (ethnicity removed from the model due to collinearity). Values <1 favour received progesterone.

^2^Adjusted for primary indication for cerclage, number of previous midtrimester losses, number of previous preterm births (<34 weeks), previous cervical surgery, cerclage technique involved bladder dissection, and suture type received (ethnicity removed from the model due to collinearity and maternal age at cerclage placement removed from the model due to convergence issues). Values <0 favour progesterone.

^3^Other includes termination due to SROM and increased risk of sepsis.

^4^Other includes a delivery <20 weeks, unknown if baby was born alive and subsequently died (neonatal death <7 days) or died prior to delivery (miscarriage).

**Table 4:** Sensitivity analysis of pregnancy loss in the C-STICH population with and without progesterone–removing terminations of pregnancy due to foetal anomaly.

	Received progesterone		Risk ratio^1^ (95% CI)	Risk difference^2^ (95% CI)
	No (*N* = 1,109)	Yes (*N* = 834)		
Pregnancy loss-*N* (%)				
Yes	85 (7.7)	48 (5.8)	0.74 (0.53, 1.05)	−0.02 (−0.04, 0.004)
No	1,018 (92.3)	784 (94.2)		
Missing	6	2		
Type of pregnancy loss-*N* (%)				
Miscarriage	48 (4)	22 (3)	–	–
Spontaneous	45	20		
Missed	1	1		
Septic	2	1		
Termination	10 (1)	3 (<1)		
Maternal medical condition	1	0		
Maternal sepsis	7	1		
Other^3^	2	2		
Stillbirth	8 (1)	10 (1)		
Other^4^	1 (<1)	0 (-)		
Neonatal death <7 days	18 (2)	13 (2)		

Pregnancy loss (sensitivity analysis of primary outcome) presented by progesterone group including adjusted risk ratio with 95% confidence intervals and adjusted risk difference with 95% confidence intervals. Progesterone use by pregnancy loss type is also presented without further analysis. Abbreviation: CI:confidence interval.

^1^Adjusted for primary indication for cerclage, number of previous midtrimester losses, number of previous preterm births (<34 weeks), cerclage technique involved bladder dissection, and suture type received and maternal age at cerclage placement (ethnicity and previous cervical surgery removed from the model due to collinearity). Values <1 favour received progesterone.

^2^Adjusted for primary indication for cerclage, number of previous midtrimester losses and number of previous preterm births (<34 weeks) (ethnicity and previous cervical surgery removed from the model due to collinearity and maternal age at cerclage placement, suture type received and cerclage technique involved bladder dissection removed from the model due to convergence issues). Values <0 favour progesterone.

^3^Other includes termination due to SROM and increased risk of sepsis.

^4^Other includes a delivery <20 weeks, unknown if baby was born alive and subsequently died (neonatal death <7 days) or died prior to delivery (miscarriage).

**Fig 1:**
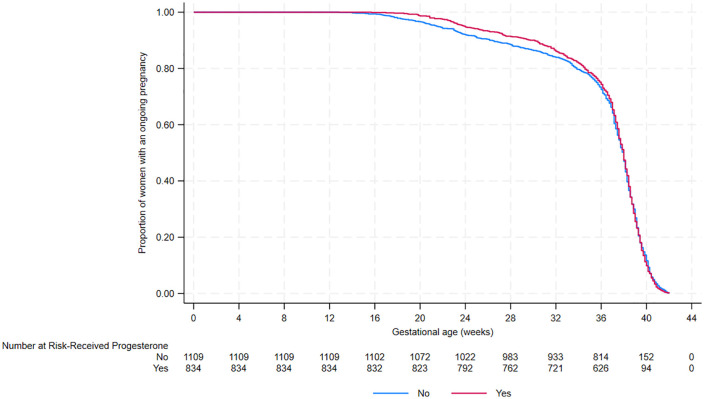
Kaplan–Meier survival plot for time from conception to pregnancy end by progesterone use.

For the key secondary analysis of time from conception to live birth (considered in a competing risk framework), women who received progesterone had a higher incidence of live birth (sub distribution hazard ratio 1.12 (95% CI [1.02, 1.23]) than women who did not receive progesterone, after accounting for the competing risk of pregnancy loss. However, these live births appeared to occur at a marginally faster rate (cause-specific hazard ratio 1.07 (95% CI [0.97, 1.18])) (Table 5). The higher incidence of live birth occurs mainly due to progesterone reducing second trimester miscarriage and previable neonatal death.For time from conception to pregnancy end, there was no evidence that receiving progesterone shortens or prolongs pregnancy duration (hazard ratio 1.04 (95% CI [0.95, 1.14], Table 7 and Fig 1).

In regards to the other maternal and neonatal secondary outcomes as reported in Table 5, the model estimates were not clinically meaningful, and the 95% CIs included the possibility of no difference between the groups. There was no evidence of varying effects in the prespecified subgroup analyses (Table 6).

**Table 5:** Secondary maternal and neonatal outcomes.

	Received progesterone		Estimate (95% CI)
	No (*N* = 1,109)	Yes (*N* = 834)	
Miscarriage and previable neonatal death-*N* (%)	63 (5.7)	35 (4.2)	0.72 (0.48, 1.08)^1^
Missing	6	2	−0.01 (−0.03, 0.005)^2^
Stillbirth-*N* (%)	8 (0.7)	10 (1.2)	1.85 (0.73, 4.70)^3^
Missing	6	2	0.005 (−0.004, 0.01)^4^
Gestational age at delivery^*^ (weeks)-Mean (SD, N)	37.2 (3.3, 1,013)	37.2 (3.3, 782)	−0.15 (−0.47, 0.16)^5^
Gestational age at delivery^*^ (<28 weeks)-*N* (%)	35 (3.5)	27 (3.5)	1.14 (0.69, 1.88)^1^ −0.002 (−0.02, 0.02)^6^
Gestational age at delivery^*^ (<32 weeks)-N (%)	85 (8.4)	66 (8.4)	1.14 (0.84, 1.56)^1^ 0.01 (−0.02, 0.03)^7^
Gestational age at delivery^*^ (<37 weeks)-N (%)	281 (27.7)	217 (27.8)	1.04 (0.89, 1.21)^8^ 0.01 (−0.03, 0.05)^9^
PPROM-*N* (%)	222 (20.0)	160 (19.2)	0.97 (0.81, 1.17)^10^
Missing	0	1	−0.002 (−0.04, 0.03)^11^
Maternal sepsis-*N* (%)	61 (5.5)	39 (4.7)	0.84 (0.57, 1.26)^1^
Missing	6	7	−0.01 (−0.03, 0.01)^12^
Early neonatal death^*^ (<7 days)-*N* (%)	4 (0.4)	4 (0.5)	1.22 (0.29, 5.08)^3^
Missing	6	2	0.001 (−0.01, 0.01)^13^
Late neonatal death^*^ (≥7 and <28 days)-*N* (%)	1 (0.1)	0 (-)	Not estimable
Missing	6	2	
Neonatal sepsis^*^ (clinically diagnosed)-*N* (%)	109 (10.9)	99 (12.8)	1.23 (0.95, 1.60)^1^
Missing	9	9	0.02 (−0.01, 0.05)^14^
Neonatal sepsis^*^ (clinically confirmed)-*N* (%)	16 (1.6)	17 (2.2)	1.59 (0.81, 3.13)^1^
Missing	10	9	0.01 (−0.01, 0.02)^15^
Time from conception to live birth^**^ (weeks)-Median [IQR]	38.1 [36.5–39.3]	38.1 [36.6–39.1]	1.12 (1.02, 1.23)^16^ 1.07 (0.97, 1.18)^17^

Secondary maternal and neonatal outcomes comparing progesterone use with no progesterone use. Effect estimates are presented with 95% confidence intervals. Abbreviations: CI: confidence interval; IQR: interquartile range; SD: standard deviation; PPROM: Preterm prelabour rupture of membranes.

* In live births ≥24 weeks (did not receive progesterone *N* = 1,013, received progesterone *N* = 782).

** In women who had a live birth surviving at least 7 days (did not receive progesterone *N* = 1,012, received progesterone *N* = 783).

^1^Risk ratio adjusted for primary indication for cerclage, number of previous midtrimester losses, number of previous preterm births (<34 weeks), cerclage technique involved bladder dissection, suture type received and maternal age at cerclage placement (ethnicity and previous cervical surgery removed from the model due to collinearity). Values <1 favour received progesterone.

^2^Risk difference adjusted for primary indication for cerclage only (ethnicity and previous cervical surgery removed from the model due to collinearity and number of previous midtrimester losses, number of previous preterm births (<34 weeks), cerclage technique involved bladder dissection, suture type received and maternal age at cerclage placement removed from the model due to convergence issues). Values <0 favour progesterone.

^3^Risk ratio adjusted for number of previous midtrimester losses, number of previous preterm births (<34 weeks), cerclage technique involved bladder dissection, suture type received and maternal age at cerclage placement (primary indication for cerclage, ethnicity and previous cervical surgery removed from the model due to collinearity). Values <1 favour received progesterone.

^4^Risk difference adjusted for number of previous midtrimester losses and number of previous preterm births (<34 weeks) (primary indication for cerclage, ethnicity and previous cervical surgery removed from the model due to collinearity and cerclage technique involved bladder dissection, suture type received and maternal age at cerclage placement removed from the model due to convergence issues). Values <0 favour progesterone.

^5^Mean difference adjusted for primary indication for cerclage, number of previous midtrimester losses, number of previous preterm births (<34 weeks), cerclage technique involved bladder dissection, suture type received, maternal age at cerclage placement, ethnicity, previous cervical surgery and gestational age at cerclage placement. Values >0 favour received progesterone.

^6^Risk difference adjusted for gestational age at cerclage placement only (ethnicity and previous cervical surgery removed from the model due to collinearity and primary indication for cerclage, number of previous midtrimester losses, number of previous preterm births (<34 weeks), cerclage technique involved bladder dissection, suture type received and maternal age at cerclage placement from the model due to convergence issues). Values <0 favour progesterone.

^7^Risk difference adjusted for gestational age at cerclage placement, primary indication for cerclage, number of previous midtrimester losses and number of previous preterm births (<34 weeks) (ethnicity and previous cervical surgery removed from the model due to collinearity and cerclage technique involved bladder dissection, suture type received and maternal age at cerclage placement, removed from the model due to convergence issues). Values <0 favour progesterone.

^8^Risk ratio adjusted for primary indication for cerclage, number of previous midtrimester losses, number of previous preterm births (<34 weeks), cerclage technique involved bladder dissection, suture type received, maternal age at cerclage placement, ethnicity, previous cervical surgery and gestational age at cerclage placement. Values <1 favour received progesterone.

^9^Risk difference adjusted for primary indication for cerclage, number of previous midtrimester losses, number of previous preterm births (<34 weeks), cerclage technique involved bladder dissection, suture type received, maternal age at cerclage placement, ethnicity, previous cervical surgery and gestational age at cerclage placement. Values <0 favour received progesterone.

^10^Risk ratio adjusted for primary indication for cerclage, number of previous midtrimester losses, number of previous preterm births (<34 weeks), cerclage technique involved bladder dissection, suture type received, ethnicity and maternal age at cerclage placement (previous cervical surgery removed from the model due to collinearity). Values <1 favour received progesterone.

^11^Risk difference adjusted for primary indication for cerclage, number of previous midtrimester losses, number of previous preterm births (<34 weeks), cerclage technique involved bladder dissection, suture type received, ethnicity and maternal age at cerclage placement (previous cervical surgery removed from the model due to collinearity). Values <0 favour received progesterone.

^12^Risk difference adjusted for primary cerclage, number of previous midtrimester losses, number of previous preterm births (<34 weeks), cerclage technique involved bladder dissection and suture type received (ethnicity and previous cervical surgery removed from the model due to collinearity and maternal age at cerclage placement removed from the model due to convergence issues). Values <0 favour received progesterone.

^13^Risk difference adjusted for number of previous midtrimester losses only (ethnicity, primary indication for cerclage and previous cervical surgery removed from the model due to collinearity and number of previous preterm births (<34 weeks), cerclage technique involved bladder dissection, suture type received and maternal age at cerclage placement removed from the model due to convergence issues). Values <0 favour received progesterone.

^14^Risk difference adjusted for primary indication for cerclage, number of previous midtrimester losses, number of previous preterm births (<34 weeks), cerclage technique involved bladder dissection, suture type received and maternal age at cerclage placement (ethnicity and previous cervical surgery removed from the model due to collinearity). Values <0 favour received progesterone.

^15^Unadjusted risk difference (ethnicity and previous cervical surgery removed from the model due to collinearity, all other covariates removed due to convergence issues). Values <0 favour received progesterone.

^16^Subdistribution hazard ratio adjusted for primary indication for cerclage, number of previous midtrimester losses, number of previous preterm births (<34 weeks), previous cervical surgery, cerclage technique involved bladder dissection, suture type received, ethnicity, maternal age at cerclage placement and gestational age at cerclage placement. Values >1 favour progesterone.

^17^Cause-specific hazard ratio adjusted for primary indication for cerclage, number of previous midtrimester losses, number of previous preterm births (<34 weeks), previous cervical surgery, cerclage technique involved bladder dissection, suture type received, ethnicity, maternal age at cerclage placement and gestational age at cerclage placement. Values <1 favour progesterone.

**Table 6:** Subgroup analyses for the primary outcome of pregnancy loss.

	Received progesterone		Interaction *p*-value	Risk ratio (95% CI)	Ratio^3^ (95% CI)
	No	Yes			
Number of previous midtrimester losses-*n/N* (%)					
<3	88/1066 (8.3)	45/806 (5.6)	0.28	0.67 (0.47, 0.96)^1^	REFERENCE
≥3	3/37 (8.1)	4/26 (15.4)		1.58 (0.36, 6.98)^1^	2.34 (0.51, 10.82)^4^
Number of previous preterm births (<34 weeks)-*n/N* (%)					
0	60/723 (8.3)	38/580 (6.6)	0.27	0.81 (0.54, 1.20)^2^	REFERENCE
≥1	31/380 (8.2)	11/252 (4.4)		0.53 (0.27, 1.02)^2^	0.65 (0.30, 1.40)^5^

Risk of pregnancy loss with and without progesterone is presented for two sub group analyses; number of previous miscarriages and number of previous preterm births. Abbreviation: CI: confidence interval.

^1^Adjusted for primary indication for cerclage, number of previous midtrimester losses, number of previous preterm births (<34 weeks), previous cervical surgery, cerclage technique involved bladder dissection, suture type received and maternal age at cerclage placement (ethnicity removed from the model due to collinearity). Values <1 favour received progesterone.

^2^Adjusted for primary indication for cerclage, number of previous midtrimester losses, number of previous preterm births (<34 weeks), previous cervical surgery, cerclage technique involved bladder dissection, suture type received and maternal age at cerclage placement (ethnicity removed from the model due to collinearity and age removed from the model due to convergence issues). Values <1 favour progesterone.

^3^Ratio of subgroup effects.

^4^Number of previous mid trimester losses (≥3) vs. number of previous mid trimester losses (<3). ^5^Number of previous pre-term births (≥1) vs. number of previous pre-term births (0).

**Table 7:** Time from conception to pregnancy end.

	Received progesterone		Adjusted hazard ratio^1^ (95% CI)
	No (*N* = 1,109)	Yes (*N* = 834)	
Time from conception to pregnancy end^2^ (weeks)			
Median [IQR, N]	38.0 [35.7–39.1, 1,109]	38.0 [36.0–39.1, 833]	1.04 (0.95, 1.14)
Minimum–Maximum	12.9–42.0	15.7–42.0	

The median time to pregnancy end is presented with and without progesterone use. There was no difference in the adjusted hazard ratio between progesterone groups. Abbreviations: CI: confidence interval; IQR: interquartile range.

^1^Adjusted for primary indication for cerclage, number of previous midtrimester losses, number of previous preterm births (<34 weeks), previous cervical surgery, cerclage technique involved bladder dissection, suture type received, ethnicity, maternal age at cerclage placement and gestational age at cerclage placement. Values <1 favour progesterone.

^2^In women with complete delivery data. Women with missing delivery data are censored in the Kaplan–Meier plot below at the point of last contact during pregnancy.

## Discussion

In women undergoing a vaginal cervical cerclage due to an increased risk of preterm birth, the addition of progesterone was associated with a 30% relative reduction in pregnancy loss. This should be interpreted considering the uncertainty within the estimates and thus the reduction could be from 1% to 50%.

The finding that progesterone potentially reduces pregnancy loss from 8.3% to 5.9% is clinically meaningful. This is in keeping with a similar analysis of 699 women by Tolosa and colleagues where perinatal death was decreased from 16% in the cerclage group to 7% in the combined cerclage and progesterone group AOR 0.37 95% CI: (0.20–0.67) [6]. This study supports the hypothesis that the use of vaginal progesterone therapy in addition to vaginal cervical cerclage is potentially associated with a reduction in pregnancy loss and therefore this may be an important advancement in the prevention of pregnancy loss. The reduction in pregnancy loss particularly relates to the reduction in second trimester miscarriage and previable neonatal death. Second trimester miscarriage is a multifactorial clinical condition and represents a likely continuum from first trimester miscarriage and it is therefore likely that progesterone is exerting its benefit primarily through this linked, but not fully understood, mechanism [14,15]. There is emerging evidence that progesterone exerts an anti-inflammatory effect, whilst cerclage provides mechanical support to the cervix and prevents cervical shortening it is reasonable to consider their use potentially complementary in improving outcomes for women [14]. Prematurity is the leading cause of perinatal and all-cause mortality for children under 5 years [16]. Therefore, while research needs to identify new ways to identify and treat those at risk, it is also important to maximise the benefit from existing therapies and identify in which women these therapies are effective. We highlight an important combination of interventions, which together appear to provide benefit. To address the limitations of this study and the potential unknown confounders within C-STICH dataset it would be important to consider how we could better understand the women who would benefit from singular and combinations of treatments of progesterone and cerclage. It is likely that further randomised controlled trials would be difficult to recruit to and clinical equipoise variable. Therefore, further evaluation in identifying when combination treatment is likely to be beneficial could be evaluated through an IPD meta-analysis. C-STICH was a large UK based randomised controlled trial with recruitment in 75 maternity units. The results presented in this manuscript are from a secondary analysis of the women within the trial and include a large number of women. The strengths of the cohort are thus the size of the cohort, the generalisability to the population of women in the UK receiving a vaginal cervical cerclage and the number of potential confounders and the robustness of the data that was collected. Limitations are the pragmatic nature of the trial and the possibility of unknown confounders that we could not account for in the statistical analysis. For example, there was variation in how progesterone was utilised across the sites. Most women commenced combination treatment around the time of cerclage placement suggesting that the history, clinical findings and the results of an ultrasound scan supported the decision for combined therapy to prevent pregnancy loss and prematurity. Approximately 25% of the cohort started progesterone treatment before the placement of a cervical cerclage suggesting a further change in clinical condition perhaps through continued shortening of the cervical length resulted in the placement of a cerclage and 39% of women commenced progesterone after placement of the cervical cerclage for presumed continued concern regarding the risk of pregnancy loss and prematurity. It could be extrapolated that the group who received combined treatment were considered to be at higher risk of a poor outcome but despite this combination treatment improved pregnancy loss rates overall. It is therefore plausible that this analysis underestimates the effect that progesterone might have had. The unexpected imbalance in rates of termination of pregnancy for foetal anomalies between the progesterone and nonprogesterone groups, is expected to have occurred due to random chance, resulted in a post-hoc analysis in which their reduction in adjusted risk ratio for pregnancy loss was not statistically significant.

There are also known variations within the cervical cerclage technique as reported in the trial results paper. Clinicians were able to determine the type of vaginal cervical cerclage that was inserted (high with bladder dissection or low cervical) based on history, ultrasound or examination findings or personal preference. Women who received progesterone were more likely to have a high vaginal cerclage involving bladder dissection. Our analysis was adjusted for cerclage technique, but it should be recognised that this is likely to reflect variation within care recommendations across the preterm birth network with some sites offering as standard a high vaginal cerclage and combination treatment with progesterone.

The C-STICH cohort represents the largest data set of women receiving cerclage and progesterone to date. In the C-STICH cohort, the addition of vaginal progesterone therapy for women undergoing vaginal cervical cerclage was associated with a 30% relative reduction in the risk of pregnancy loss. There is a need for further evidence to determine the women most likely to benefit from combined cerclage and progesterone therapy, the timing and dosage of progesterone therapy in women undergoing a cervical cerclage and the true effect size.

## Supporting information

S1 Checklist STROBE checklist. STROBE Statement for the secondary analysis of C-STICH explore concomitant cervical cerclage and progesterone [17].
(DOCX)

S1 Text The C-STICH study: a secondary analysis to examine the effect of cervical cerclage in combination with progesterone. (DOCX)

## Abbreviations

BOLD: blood-oxygen-level-dependent
CI: confidence interval
HR: hazard ratio
PC: piriform cortex
PPROM: preterm prelabour rupture of membranes
RD: risk difference
RR: risk ratio
