# Supplementary material for: Pregnancy outcomes in women at high risk of preterm birth receiving a vaginal cervical cerclage with, or without, progesterone: A retrospective, secondary analysis of the C-STICH randomised controlled trial data
Source: PLoS Med. 2026 Jan 23;23(1):e1004513. doi: 10.1371/journal.pmed.1004513 (PMC12872024; doi:10.1371/journal.pmed.1004513)
Supplement: S1 Text — (DOCX) [file pmed.1004513.s002.docx]

**The C-STICH study: A secondary analysis to examine the effect of cervical cerclage in combination with progesterone**

**Statistical Analysis Plan**

| SAP Version Number |
| --- |
| 1.0 |

| Author: | Catherine Moakes | Role: | Senior Statistician | Affiliation: | BCTU  University of Birmingham |
| --- | --- | --- | --- | --- | --- |
| Signature: |  | Date: |  |  |  |
| Lead Investigator: | Vicky Hodgetts Morton | Role: | Lead Investigator | Affiliation: | Birmingham Women’s and Children’s |
| Signature: |  | Date: |  |  |  |
| **This Statistical Analysis Plan has been approved by:** | | | | | |
| Approver: | Lee Middleton | Role: | Senior Statistician | Affiliation: | BCTU  University of Birmingham |
| Signature: |  | Date: |  |  |  |

Table 1: SAP Amendments

| **SAP version number** | **Date Approved** | **Protocol version number†** | **Section number changed** | **Description of and reason for change** | **Timing of change with respect to interim/final analysis** | **Blind Reviewer** |
| --- | --- | --- | --- | --- | --- | --- |
| **NA** | | | | | | |

| **Abbreviations & Definitions** | |
| --- | --- |
| **Abbreviation/Acronym** | **Meaning** |
| BCTU | Birmingham Clinical Trials Unit |
| CI | Confidence Interval |
| EDD | Expected Date of Delivery |
| NHS | National Health Service |
| PPROM | Preterm pre labour rupture of membranes |
| RD | Risk Difference |
| RR | Relative Risk/Risk Ratio |
| SAE | Serious Adverse Event |
| SAP | Statistical Analysis Plan |
| UK | United Kingdom |
| **Term** | **Definition** |
| Statistical Analysis Plan | Pre-specified statistical methodology documented for the study, either in the protocol or in a separate document. |

**TABLE OF CONTENTS**

[**1.** **Introduction** 6](#_Toc143603333)

[**2.** **Background and rationale** 6](#_Toc143603334)

[**3.** **Study objectives** 6](#_Toc143603335)

[**4.** **Methods** 6](#_Toc143603336)

[4.1. Study design 6](#_Toc143603337)

[4.2. Primary outcome measure 6](#_Toc143603338)

[4.3. Secondary outcome measures 6](#_Toc143603339)

[4.4. Timing of outcome assessments 7](#_Toc143603340)

[4.5. Randomisation 7](#_Toc143603341)

[4.6. Sample size 7](#_Toc143603342)

[4.7. Study comparisons 7](#_Toc143603343)

[**5.** **Statistical Principles** 7](#_Toc143603344)

[5.1. Confidence intervals and p-values 7](#_Toc143603345)

[5.2. Adjustments for multiplicity 7](#_Toc143603346)

[5.3. Analysis populations 7](#_Toc143603347)

[**6.** **Study population** 7](#_Toc143603348)

[6.1. Recruitment 7](#_Toc143603349)

[6.2. Baseline characteristics 7](#_Toc143603350)

[**7.** **Intervention** 7](#_Toc143603351)

[7.1. Description of the intervention 7](#_Toc143603352)

[**8.** **Analysis methods** 7](#_Toc143603353)

[8.1. Covariate adjustment 8](#_Toc143603354)

[8.2. Distributional assumptions 8](#_Toc143603355)

[8.3. Handling missing data 9](#_Toc143603356)

[8.4. Data manipulations 9](#_Toc143603357)

[8.5. Analysis methods – primary outcome 9](#_Toc143603358)

[8.6. Analysis methods – secondary outcomes 9](#_Toc143603359)

[8.7. Planned subgroup analyses 9](#_Toc143603360)

[8.8. Sensitivity analyses or supportive analyses 9](#_Toc143603361)

[**9.** **Statistical software** 10](#_Toc143603362)

[**10.** **References** 10](#_Toc143603363)

[**Appendix A: Data Manipulations** 11](#_Toc143603364)

| **Introduction** |
| --- |
| This document is the Statistical Analysis Plan (SAP) for the secondary analysis of the C-STICH trial. This SAP details the proposed analyses for the main paper(s) reporting the results for this secondary analysis.  The results reported in these papers will follow the strategy set out here. Subsequent analyses of a more exploratory nature will not be bound by this strategy, though they are expected to follow the broad principles laid down here. The principles are not intended to curtail exploratory analysis (e.g. to decide cut-points for categorisation of continuous variables), nor to prohibit accepted practices (e.g. transformation of data prior to analysis), but they are intended to establish rules that will be followed, as closely as possible, when analysing and reporting data.  The analysis will be carried out by an appropriately qualified statistician, who should ensure integrity of the data. |
| **Background and rationale** |
| In brief, C-STICH was a multi-centre, open, randomised controlled trial of 2049 women presenting at obstetric units and deemed to be at risk of an insufficient cervix, and scheduled to be treated by cervical cerclage [1] This secondary analysis of the C-STICH trial data will examine the effect of cervical cerclage in combination with progesterone on outcomes collected and reported in the C-STICH trial. |
| **Study objectives** |
| The primary objective is to examine the effect of receiving progesterone compared with not receiving progesterone on pregnancy loss in women deemed to be at risk of an insufficient cervix and treated with cervical cerclage.  Secondary objectives are as follows:   - To assess the characteristics of women (including indication for cerclage) for those who receive or do not receive progesterone. - To assess the effect of progesterone on other pregnancy and neonatal outcomes. |
| **Methods** |
| Study design |
| C-STICH was a multicentre, open, superiority, parallel-group, randomised controlled trial. Women were recruited from National Health Service (NHS) obstetric and gynaecology units in the United Kingdom (UK) and Ireland. This secondary analysis will be an observational study assessing outcomes in women who received a cervical cerclage +/- progesterone. |
| Primary outcome measure |
| Pregnancy loss rate (miscarriage and perinatal mortality, including any stillbirth or neonatal death in the first week of life). |
| Secondary outcome measures |
| A key secondary outcome is time from conception to pregnancy end (any reason).  Other maternal and neonatal secondary outcomes include:  Maternal   - Miscarriage & previable neonatal death (defined as delivery <24 weeks). - Stillbirth (defined as intrauterine death ≥24 weeks). - Gestational age at delivery (in live births ≥24 weeks). - Gestational age <28/<32/<37 weeks at delivery (in live births ≥24 weeks). - Preterm pre labour rupture of membranes (PPROM). - Sepsis (at any time in pregnancy and until 7 days postnatal).   Neonatal   - Early neonatal death (defined as a death within 7 days after delivery, in live births ≥24 weeks). - Late neonatal death (defined as a death beyond 7 days and before 28 days after delivery, in live births ≥24 weeks). - Sepsis (clinically diagnosed/proven) (in live births ≥24 weeks). |
| Timing of outcome assessments |
| Pregnancy outcomes are measured during the antenatal and intrapartum period. Maternal outcomes are measured up to 7 days post-delivery. Neonatal outcomes are measured up to 28 days post-delivery in term neonates (≥37 weeks gestational age) and up to 28 days post expected date of delivery (EDD) in pre-term neonates (<37 weeks gestational age). |
| Randomisation |
| Not applicable this is an observational study. |
| Sample size |
| The sample size for this secondary analysis will include all women randomised to C-STICH who had a cervical cerclage placed and had available progesterone use data (N=1943). Whilst this sample size has not been specifically tailored to this secondary analysis, this size of sample would be sufficient to detect a reduction in pregnancy loss from 9.3% in the no progesterone group to 5.7% in the progesterone group [2,3] with 85% power. |
| Study comparisons |
| All references in this document to ‘group’ refer to received progesterone or no progesterone. For the purpose of this analysis and tabulations, no progesterone will be considered the control or ‘reference’ group. |
| **Statistical Principles** |
| Confidence intervals and p-values |
| All estimates of differences between groups will be presented with two-sided 95% confidence intervals (CIs), unless otherwise stated. |
| Adjustments for multiplicity |
| No correction for multiple testing will be made. |
| Analysis populations |
| All women who received a cervical cerclage with available progesterone data will be included. |
| **Study population** |
| Recruitment |
| A flow diagram (as recommended by STROBE [4]) will be produced to describe the participant flow through each stage of the study. |
| Baseline characteristics |
| The study population will be tabulated. Categorical data will be summarised by number of participants, counts and percentages. Continuous data will be summarised by the number of participants, mean and standard deviation if deemed to be normally distributed or number of participants, median and interquartile range if data appear skewed, and ranges if appropriate. Tests of statistical significance will be undertaken, using t-tests (Wilcoxon rank sum tests) for normally distributed (non-normal) continuous variables and chi-square/Fishers exact tests for categorical variables. Baseline variables include: primary indication for cerclage, number of previous mid trimester losses, number of previous pre-term births, previous cervical surgery, cerclage technique (bladder dissection), suture type received, ethnicity, maternal age at cerclage placement and gestational age at cerclage placement. |
| **Intervention** |
| Description of the intervention |
| The number and proportion of women who receive progesterone will be presented alongside the gestational age when progesterone was received. Time from cerclage placement to progesterone start date will also be computed and dichotomised as before cerclage placement, within one week of cerclage placement, and after cerclage placement. |
| **Analysis methods** |
| Intervention groups will be compared using regression models to adjust for all covariates as specified in section 8.1 where possible. |
| Covariate adjustment |
| In the first instance, intervention effects between groups will be adjusted for the following variables:   - Primary indication for cerclage (A history of three or more previous midterm losses or premature births (≤28 weeks)/Insertion of cervical sutures in previous pregnancies/A history of mid trimester loss or premature birth with a (current) shortened (≤25mm) cervix/Women whom clinicians deem to be at risk of preterm birth either by history or the results of an ultrasound scan) - Number of previous mid trimester losses - Number of previous pre-term births (<34 weeks) - Any previous cervical surgery (yes/no) - Cerclage technique involved bladder dissection (yes/no) - Suture type received (monofilament cerclage/braided cerclage) - Ethnicity (White/Black/Asian/Mixed/Other/Declined to provided information) - Maternal age at cerclage placement (years)   All covariates will be treated as fixed effects in the models. Time from conception to pregnancy end and gestational age at delivery will be further adjusted for gestational age at cerclage placement.  If full covariate adjustment is not possible (e.g. the model does not converge), covariates will be removed sequentially (in the reverse order to those listed above i.e. maternal age would be removed first) and the model refitted. If this reduced model still fails to converge, the step above will be repeated (for time from conception to pregnancy end and gestational age at delivery, gestational age at cerclage placement will be removed last). This will continue until either the model converges or until all covariates are removed and unadjusted estimates will be produced.  For binary outcomes only, if the (full) adjusted log-binomial model fails to converge, a Poisson regression model with robust standard errors will be used to estimate the risk ratio (RR) [5]. If this also fails to converge, estimates will be produced from the log-binomial model (following rules for removal of variables as outlined above).  It will be made clear why covariates were removed and which models were utilised (e.g. not possible due to low event rate/lack of model convergence). |
| Distributional assumptions |
| Distributional assumptions (e.g. normality of regression residuals for continuous outcomes) will be assessed visually prior to analysis. In the first instance the proposed primary method of estimation in this analysis plan will be followed. If responses are considered to be particularly skewed and/or distributional assumptions violated, the impact of this will be examined through sensitivity analysis. This may consist of either transformation of responses prior to analysis (e.g. log transformation) or the use of medians and interquartile ranges alongside unadjusted differences in medians using bootstrapping methods (repetition=1000, seed=123456).  For time from conception to pregnancy end, assessment of the proportional hazards assumption will be considered. This will be assessed both graphically and analytically. For graphical assessment, Kaplan Meier plots and log(-log(survival)) versus log of survival time plots will be produced. For analytical assessment, flexible parametric models will be fitted to model the log cumulative baseline hazard and time dependent effects of treatment using restricted cubic splines. For both the time dependent effects and baseline log cumulative hazard, different combinations of degrees of freedom will be fitted (0-10), where the number of degrees of freedom for the log cumulative baseline hazard is always higher than the degrees of freedom for the time dependent effects. The Bayesian information criterion (BIC) will be computed for each model. The model with the lowest BIC value will be considered the ‘best’ fit. If this model is one where the degrees of freedom for the time dependent effects are ≠ 0 then we will conclude non-proportional hazards. A plot of hazard ratio over time will be produced. |
| Handling missing data |
| In regards to adjustment covariates, indication for cerclage, received suture type, maternal age at cerclage placement, number of previous mid-trimester losses and number of previous pre-term births are complete variables (i.e. no missing data). The covariates cerclage technique involving bladder dissection, ethnicity, and previous cervical surgery have <1% of missing data. In the C-STICH trial, actual and intended cerclage technique involving bladder dissection were collected. In participants with missing information regarding actual cerclage technique involving bladder dissection (N=3), we will use data regarding intention for cerclage technique involving bladder dissection. Participants with missing ethnicity data (N=15), will form the group ‘declined to provide information’. Participants with missing data for previous cervical surgery (N=4), will form the group ‘unknown’.  In regards to outcomes, since the proportion of participants with missing data for the primary outcome is <1% (N=8) no further analyses to assess the impact of missing data will be conducted (i.e. complete case analysis only). |
| Data manipulations |
| See Appendix A: Data Manipulations. |
| Analysis methods – primary outcome |
| The primary outcome will be summarised using frequencies and percentages. A mixed effects log-binomial model will be used to generate an adjusted RR (and 95% CI). An adjusted risk difference (RD) (and 95% CI) will also be presented (using an identity link function). See section 8.1 for covariate adjustment and model convergence. |
| Analysis methods – secondary outcomes |
| For continuous secondary outcome measures (gestational age at delivery) means and standard deviations will be reported alongside an adjusted mean difference (with 95% CI) estimated using a linear regression model.  Time to event data (time from conception to pregnancy end) will be summarised using medians and interquartile ranges. A Cox regression model will be fitted to generate an adjusted hazard ratio (and 95% CI) and a Kaplan Meier plot will be produced to assess the data visually.  Binary outcomes (miscarriage & pre-viable neonatal death, stillbirth, gestational age <28/<32/<37 weeks, PPROM, early neonatal death and late neonatal death) will be analysed as per the primary outcome.  See section 8.1 for covariate adjustment and model convergence. |
| Planned subgroup analyses |
| Interpretation of subgroup analyses will be treated with caution (output will be treated as exploratory rather than definitive [6]). Analyses will be limited to the primary outcome only, and the following subgroups:   - Number of previous mid trimester losses (<3/≥3) - Number of previous pre-term births (<34 weeks) (0/≥1)   The effects of these subgroups will be examined by including a treatment group by subgroup interaction parameter in the regression model. P-values from tests for statistical heterogeneity will be presented alongside the effect estimate and 95% CI within each subgroup. In addition to this, a ratio (and 95% CI) will be provided to quantify the difference between the intervention effects estimated within each subgroup. |
| Sensitivity analyses or supportive analyses |
| Sensitivity/supportive analyses will consist of:   - An analysis to assess distributional assumptions (where applicable, described in section 8.2) for continuous secondary outcomes. |
| **Statistical software** |
| Statistical analysis will be undertaken in the following statistical software packages: Stata (version 18.0 or higher) and SAS (version 9.4 or higher). |
| **References** |
| 1. Monofilament suture versus braided suture thread to improve pregnancy outcomes after vaginal cervical cerclage (C-STICH): a pragmatic randomised, controlled, phase 3, superiority trial, Morton et al., Lancet, VOLUME 400, ISSUE 10361, P1426-1436, OCTOBER 22, 2022 2. Berghella V, Figueroa D, Szychowski JM, Owen J, Hankins GD, Iams JD, Sheffield JS, Perez-Delboy A, Wing DA, Guzman ER; Vaginal Ultrasound Trial Consortium. 17-alpha-hydroxyprogesterone caproate for the prevention of preterm birth in women with prior preterm birth and a short cervical length. Am J Obstet Gynecol. 2010 Apr;202(4):351.e1-6. doi: 10.1016/j.ajog.2010.02.019. PMID: 20350641; PMCID: PMC2855838. 3. Roman AR, Da Silva Costa F, Araujo Júnior E, Sheehan PM. Rescue Adjuvant Vaginal Progesterone May Improve Outcomes in Cervical Cerclage Failure. Geburtshilfe Frauenheilkd. 2018 Aug;78(8):785-790. doi: 10.1055/a-0637-9324. Epub 2018 Aug 20. PMID: 30140107; PMCID: PMC6102116. 4. Von Elm E, Altman DG, Egger M, Pocock SJ, Gotzsche PC, Vandenbroucke JP. The Strengthening the Reporting of Observational Studies in Epidemiology (STROBE) Statement: guidelines for reporting observational studies 5. Zou G. A modified Poisson regression approach to prospective studies with binary data. Am J Epidemiol. 2004;159(7):702-6 6. Wand R, Lagakos SW, Ware JH, Hunter DJ, Drazen JM. Reporting of subgroups analyses in clinical trials. NEJM. 2007;357:2189-94 |

| **Appendix A: Data Manipulations** |
| --- |
| The statistician will derive the following measures as follows:  Outcome measures   - **Pregnancy loss rate (miscarriage and perinatal mortality, including any stillbirth, termination of pregnancy or neonatal death in the first week of life).**   Reported on the pregnancy outcome form. If ‘baby born alive’ is no, then this will constitute meeting the primary outcome definition (i.e. pregnancy loss is yes). If ‘baby born alive’ is yes proceed as below. If on the baby outcome form ‘has the baby been discharged from hospital care?’ is yes and ‘was the baby alive when it was discharged from hospital Care’ is yes then assume baby alive at 7 days unless notified by site of known neonatal death post-discharge (within 7 days of delivery if discharged prior to this). If known neonatal loss post-discharge check time to death as per (A) below.  If ‘Has the baby been discharged from hospital care?’ is yes and ‘Was the baby alive when it was discharged from hospital Care’ is no then check time to death as follows:  (A) Time to death (days) = Date of death-Date of delivery.  If time to death <7 days then this will meet the primary outcome definition.  If time to death ≥7 this will not meet the primary outcome definition.  If ‘has the baby been discharged from hospital care’ is no then we should have >7 days of follow-up data available. Any neonates who fall into this category will be regarded as alive at 7 days (i.e. not meeting the primary outcome), unless notified by site of known neonatal death post-data collection. If known neonatal death post-data collection check time to death as per (A) above.   - **Time from conception to pregnancy end (any reason)**   Pregnancy end (miscarriage, termination, still birth or live birth) will be regarded as an event. Date of conception will be regarded as time 0 and time to pregnancy end will be derived as follows:  Time to pregnancy end (weeks) = 40-(((Expected date of delivery)-(Date of delivery))/7)  Participants will be censored if pregnancy outcome data unknown. They will be censored at the point of last known follow-up during pregnancy.   - **Miscarriage & pre-viable neonatal death (defined as delivery <24 weeks)**   Reported on the pregnancy outcome form. If ‘baby born alive’ is no and ‘cause of death’ is miscarriage (spontaneous, missed or septic) then this will meet this outcome. If ‘baby born alive’ is yes and baby is born <24 weeks, check if baby died following delivery. If baby discharged alive or baby not discharged at time of form completion assume no neonatal death unless informed by site of neonatal death post-discharge/data collection. If baby died, this will constitute meeting the outcome definition.   - **Stillbirth (defined as intrauterine death ≥24 weeks)**   Reported on the pregnancy outcome form. If ‘baby born alive’ is no and ‘cause of death’ is stillbirth this will constitute meeting the outcome definition.   - **Gestation at delivery (live births ≥24 weeks)**   Exclude any participants whose baby died (miscarriage, stillbirth or termination) prior to delivery (identified on the pregnancy outcome form). Exclude any participants who had a live birth <24 weeks. Gestation at delivery will be derived as follows:  Gestation at delivery = 40-(((Expected date of delivery)-(Date of delivery))/7)   - **Gestational age <28/<32/<37 weeks at delivery (in live births ≥24 weeks)**   Gestational age at delivery will be calculated as above and dichotomised as per thresholds outlined.   - **Preterm pre labour rupture of membranes (PPROM)**   If ‘did the mother develop preterm pre labour rupture of membranes (PPROM)’ is reported as yes on the pregnancy outcome form this will be regarded as meeting the outcome.  A composite outcome of cerclage removal complications will also be considered. If any of the above components are yes then this outcome will be yes. If all of the above components are no this will be regarded as no. All other responses will be considered missing.   - **Maternal sepsis (at any time in pregnancy and until 7 days postnatal)**   If ‘did the mother develop any sepsis within the pregnancy or within 7 days postnatal’ is reported as yes on the maternal outcome form this will be regarded as meeting the outcome.  For women who do not have postnatal follow-up of seven days, due to discharge prior to this point, if no sepsis up to the time of discharge and no SAE form for a re-admission due to sepsis, assume no sepsis at seven days postnatal.   - **Early neonatal death (defined as a death within 7 days after delivery)**   Exclude any participants whose baby died (miscarriage, still birth or termination) prior to delivery. Exclude any participants who had a live birth <24 weeks. The baby outcome form will be used to determine status of baby up to seven days. If ‘has the baby been discharged from hospital care?’ is yes and ‘was the baby alive when it was discharged from hospital Care’ is yes then assume baby alive at 7 days unless notified by site of known neonatal death post-discharge (within 7 days of delivery if discharged prior to this). If known neonatal loss post-discharge check time to death as per (A) below.  If ‘Has the baby been discharged from hospital care?’ is yes and ‘Was the baby alive when it was discharged from hospital Care’ is no then check time to death as follows:   1. Time to death (days) = Date of death-Date of delivery   If time to death <7 days then this will meet the outcome definition.  If time to death ≥7 days this will not meet the outcome definition.  If ‘has the baby been discharged from hospital care’ is no then we should have >7 days of follow-up data available. Any neonates who fall into this category will be regarded as alive at 7 days (i.e. not meeting the primary outcome), unless notified by site of known neonatal death post-data collection. If known neonatal death post-data collection check time to death as per (A) above.   - **Late neonatal death (defined as a death beyond 7 days and before 28 days after delivery)**   Exclude any participants whose baby died (miscarriage, still birth or termination) prior to delivery. Exclude any participants who had a live birth <24 weeks. The baby outcome form will be used to determine status of baby up to seven days. If ‘has the baby been discharged from hospital care?’ is yes and ‘was the baby alive when it was discharged from hospital Care’ is yes then assume baby alive at 28 days unless notified by site of known neonatal death post-discharge (within 28 days of delivery if discharged prior to this). If known neonatal loss post-discharge check time to death as per (A) below.  If ‘Has the baby been discharged from hospital care?’ is yes and ‘Was the baby alive when it was discharged from hospital Care’ is no then check time to death as follows:   1. Time to death (days) = Date of death-Date of delivery   If time to death <7 days then this will not meet the outcome definition.  If time to death ≥7 and <28 days this will meet the outcome definition.  If time to death ≥28 days this will not meet the outcome definition.  If ‘has the baby been discharged from hospital care’ is no then we should have 28 days of follow-up data available. Any neonates who fall into this category will be regarded as alive at 28 days (i.e. not meeting the primary outcome), unless notified by site of known neonatal death post-data collection. If known neonatal death post-data collection check time to death as per (A) above. Any neonates who die ≥28 days post-delivery will be reported descriptively only.   - **Neonatal sepsis (clinically diagnosed/proven) (in live births ≥24 weeks)**   Exclude any participants whose baby died (miscarriage, stillbirth or termination) prior to delivery. Exclude any participants who had a live birth <24 weeks. For clinically diagnosed sepsis, if ‘were any antibiotics administered to the baby in the 72 hours after birth’ is yes and ‘were antibiotics continued for 72 hours or more’ is yes then this will meet the outcome definition. For clinically proven sepsis, if ‘were any antibiotics administered to the baby in the 72 hours after birth’ is yes and ‘were antibiotics continued for 72 hours or more’ is yes and ‘was infection microbiologically confirmed’ is yes then this will meet the outcome definition.  Other measures   - **Gestational age at cerclage** **placement (weeks)** = 40-(((Expected date of delivery)-(Date of cerclage placement))/7). - **Maternal age at cerclage** **placement (years)** = (Date of cerclage placement-date of birth)/365.25. - **Ethnicity** - White (British, Irish, Other) - Asian (Indian, Pakistani, Bangladeshi, Chinese, Other) - Black (African, Caribbean, Other) - Mixed (White/Black Caribbean, White/Black African, White/Asian, Other) - Other (Other). - **Time from cerclage placement to progesterone start date (days)** = (Date of progesterone start-Date of cerclage placement). |
